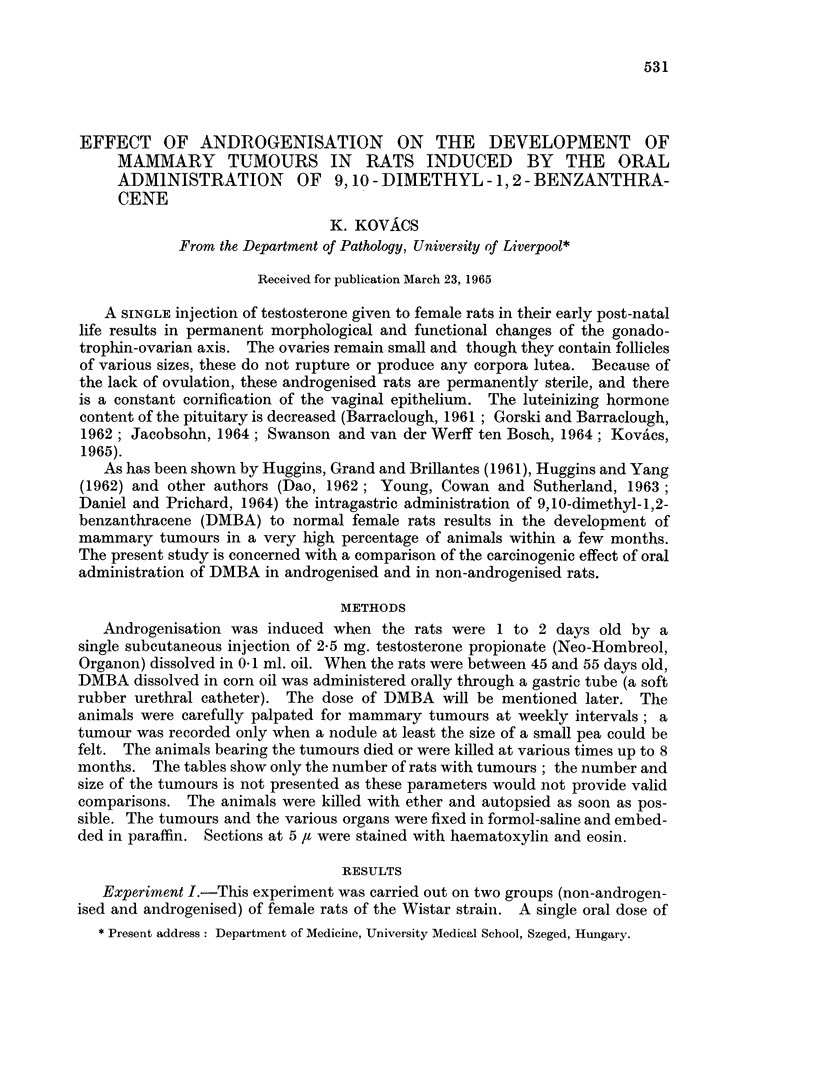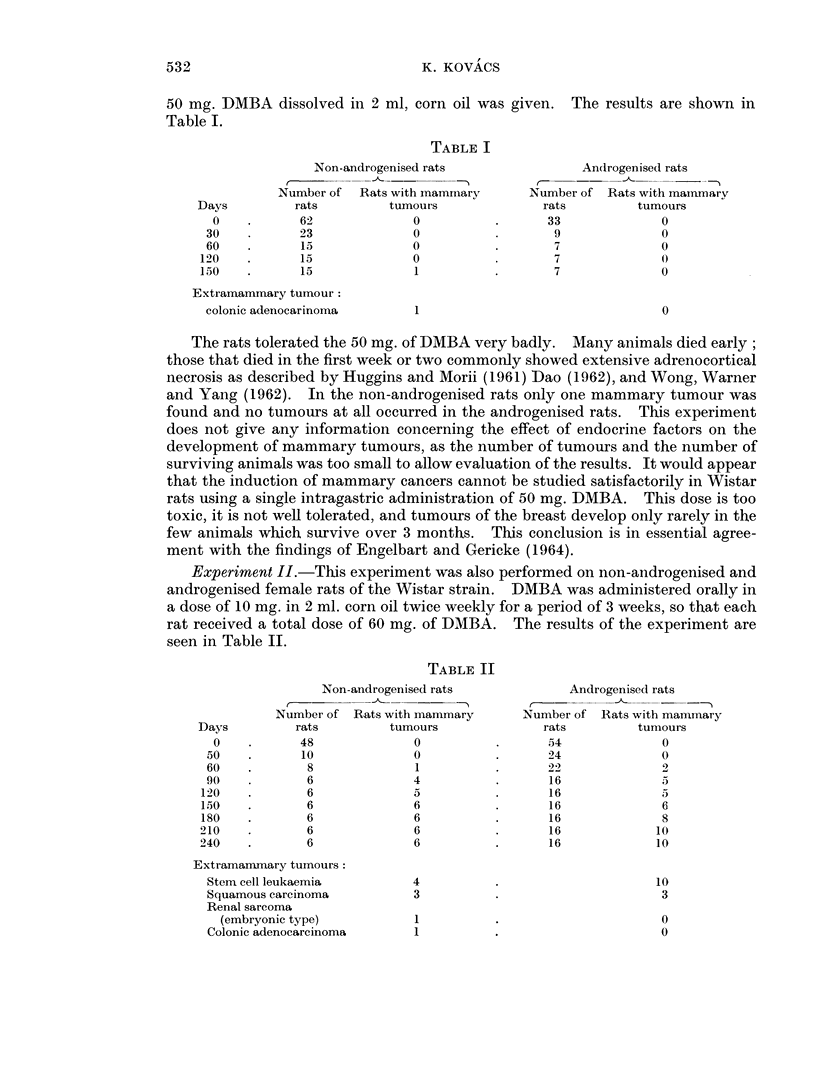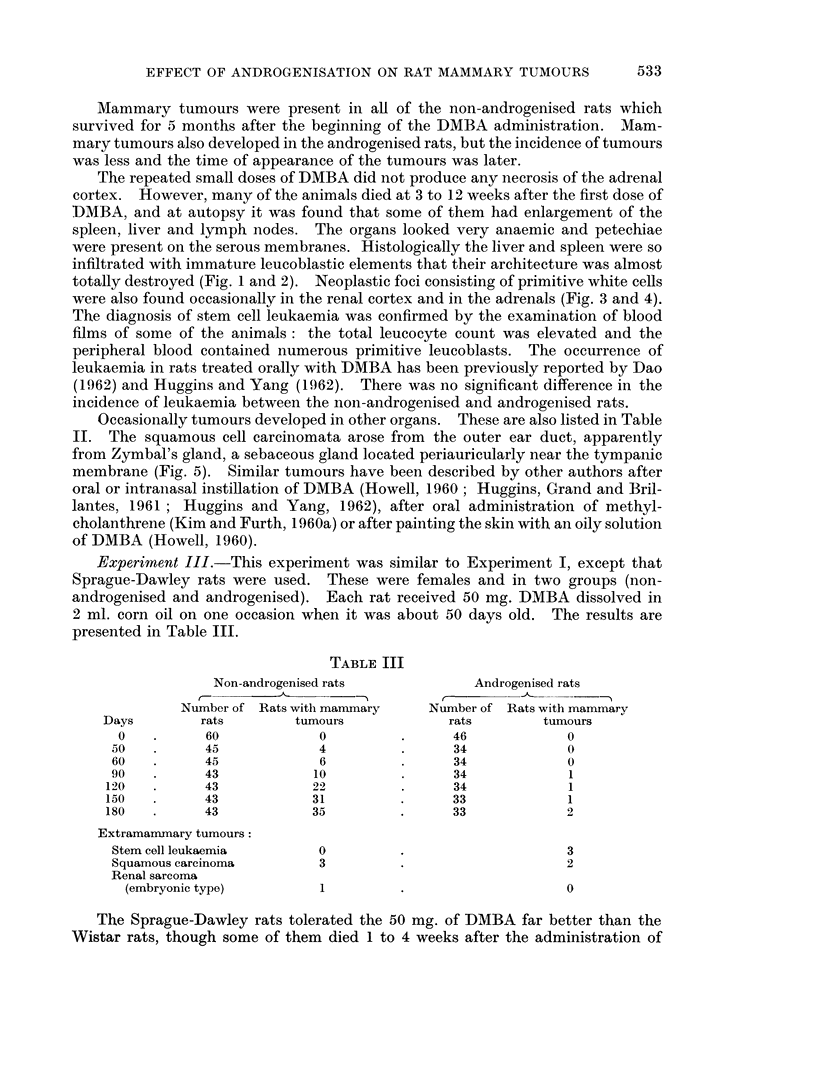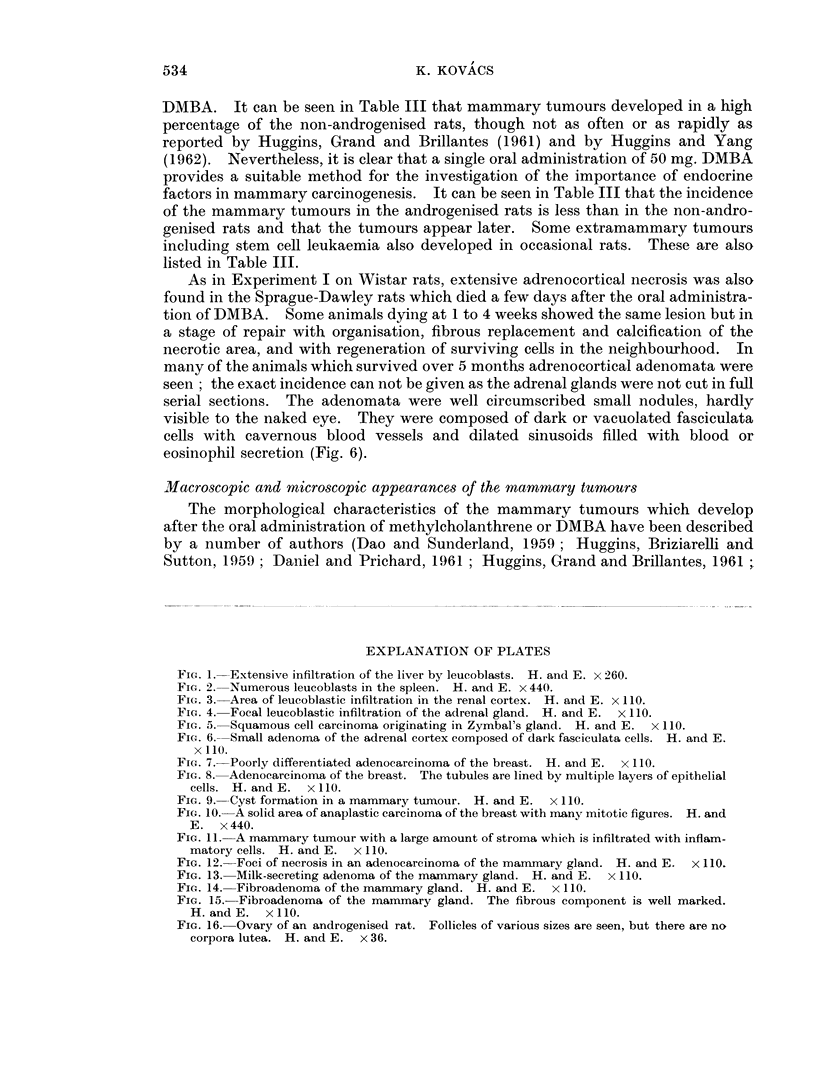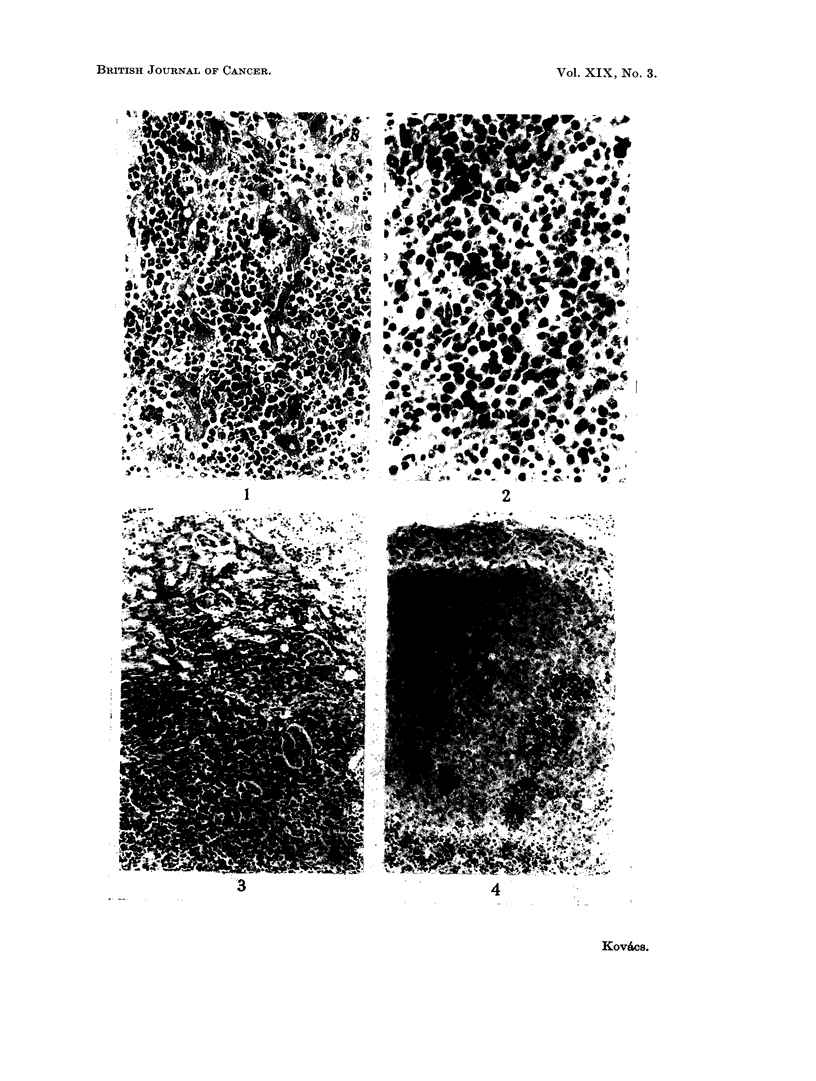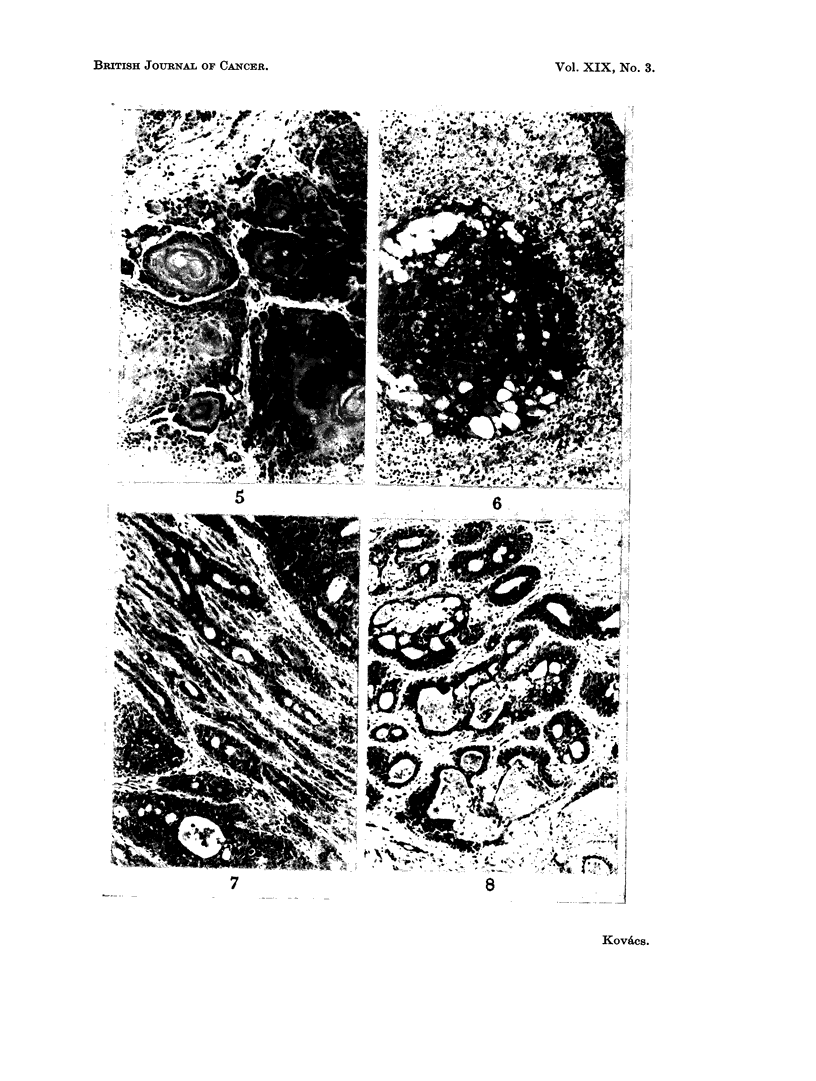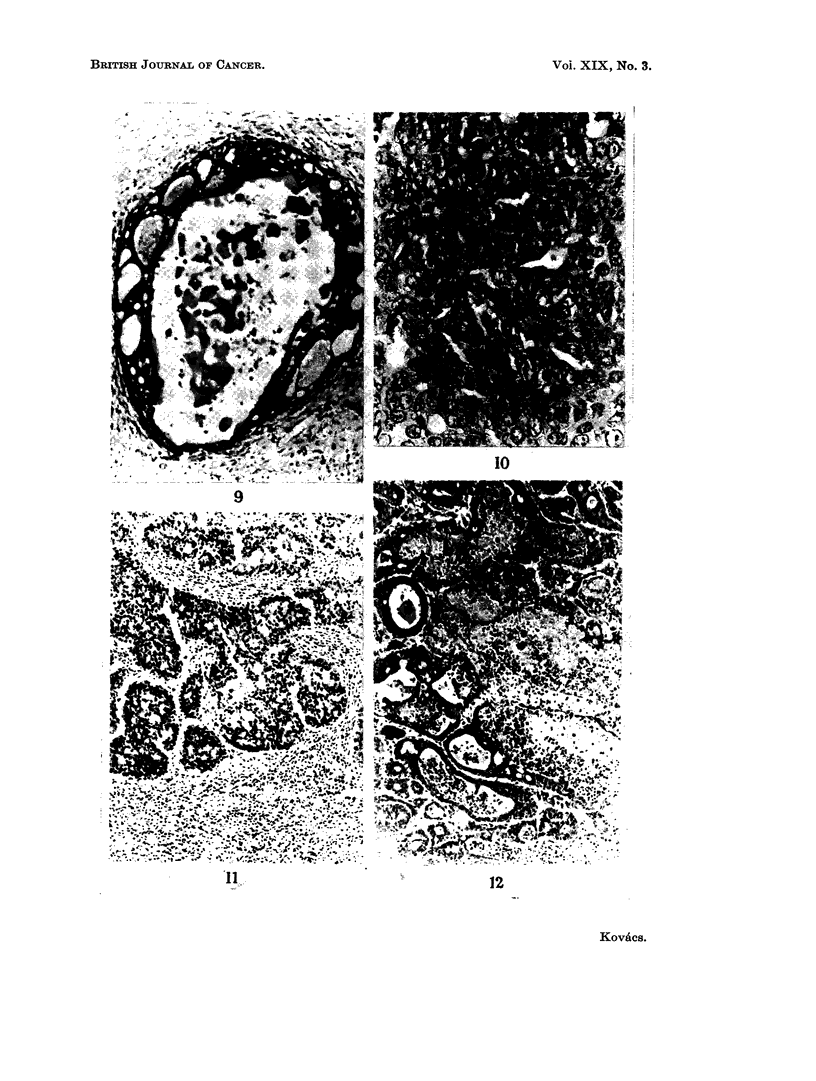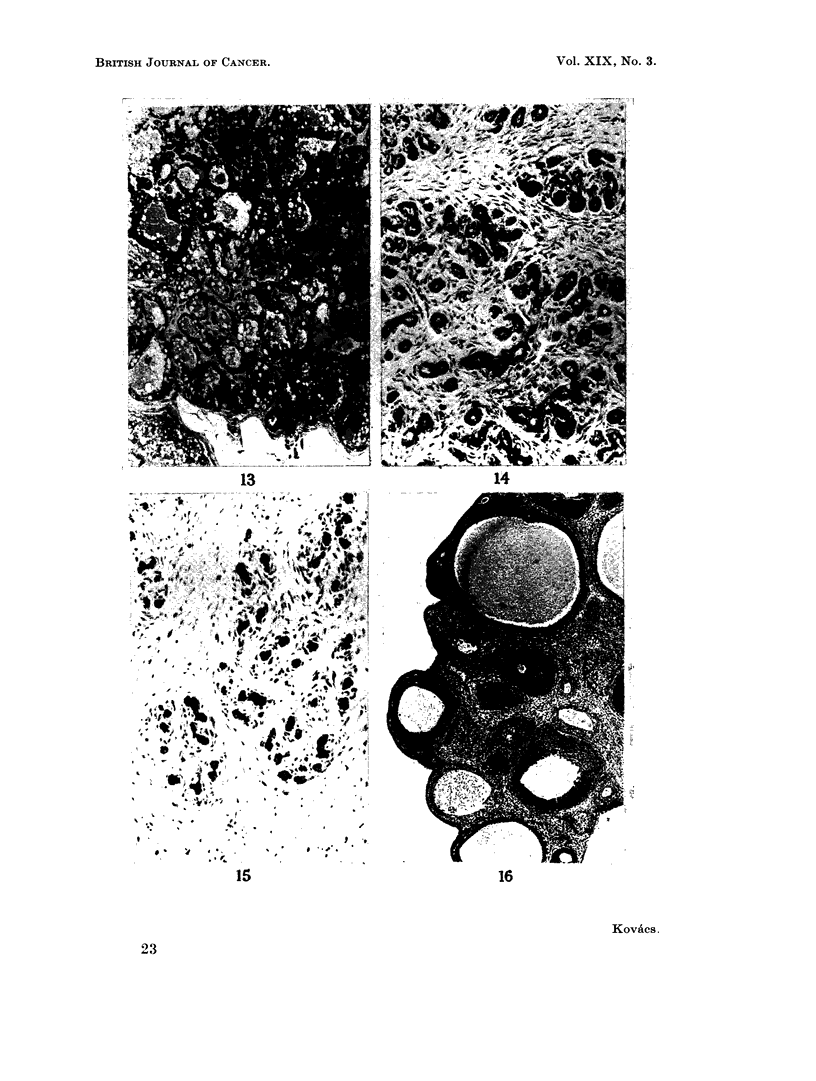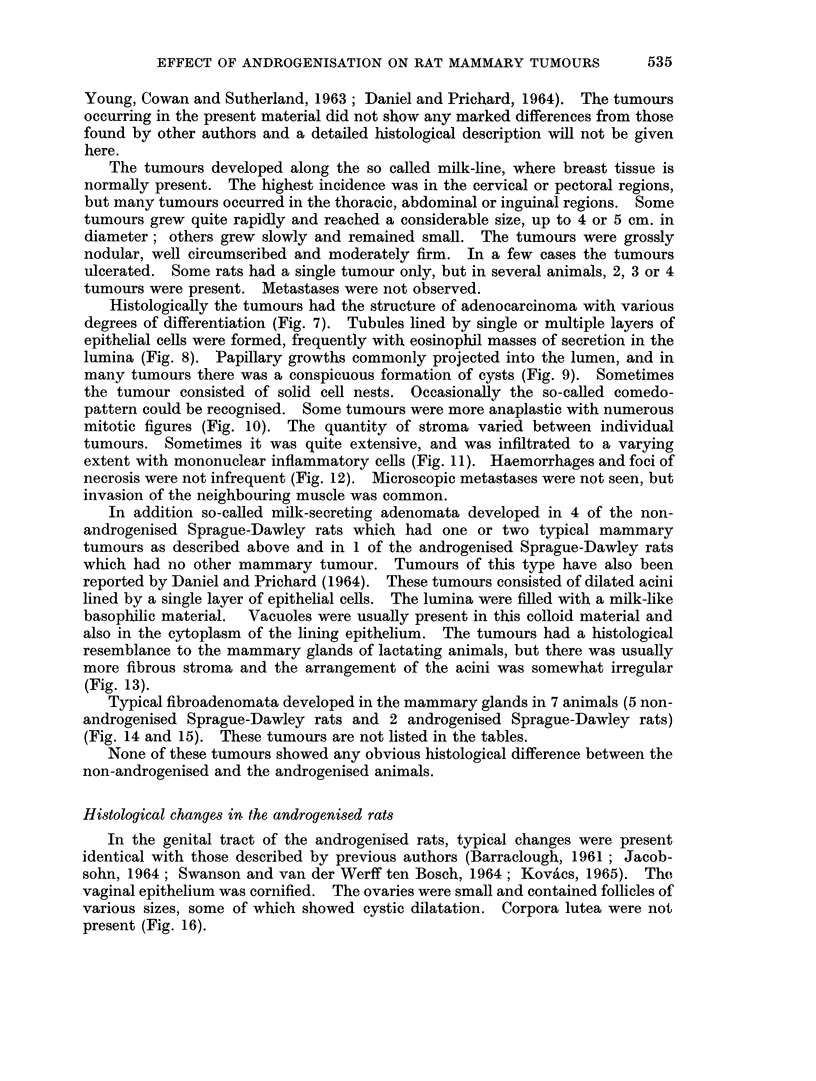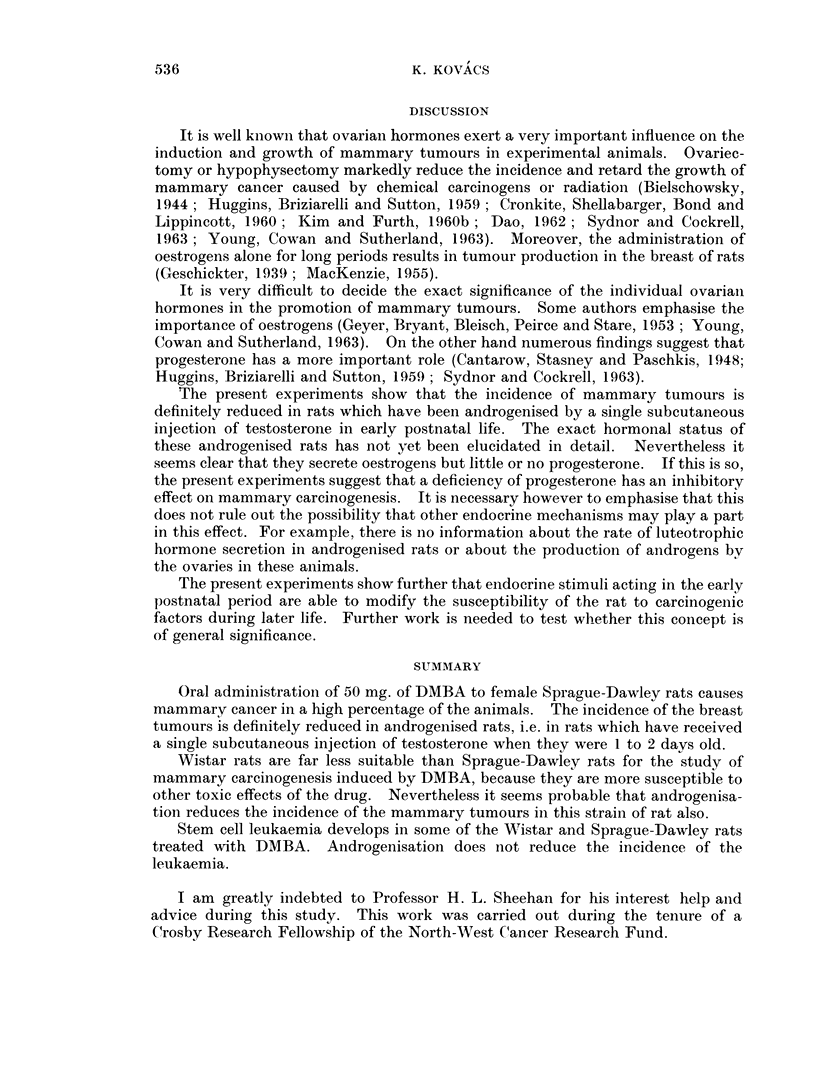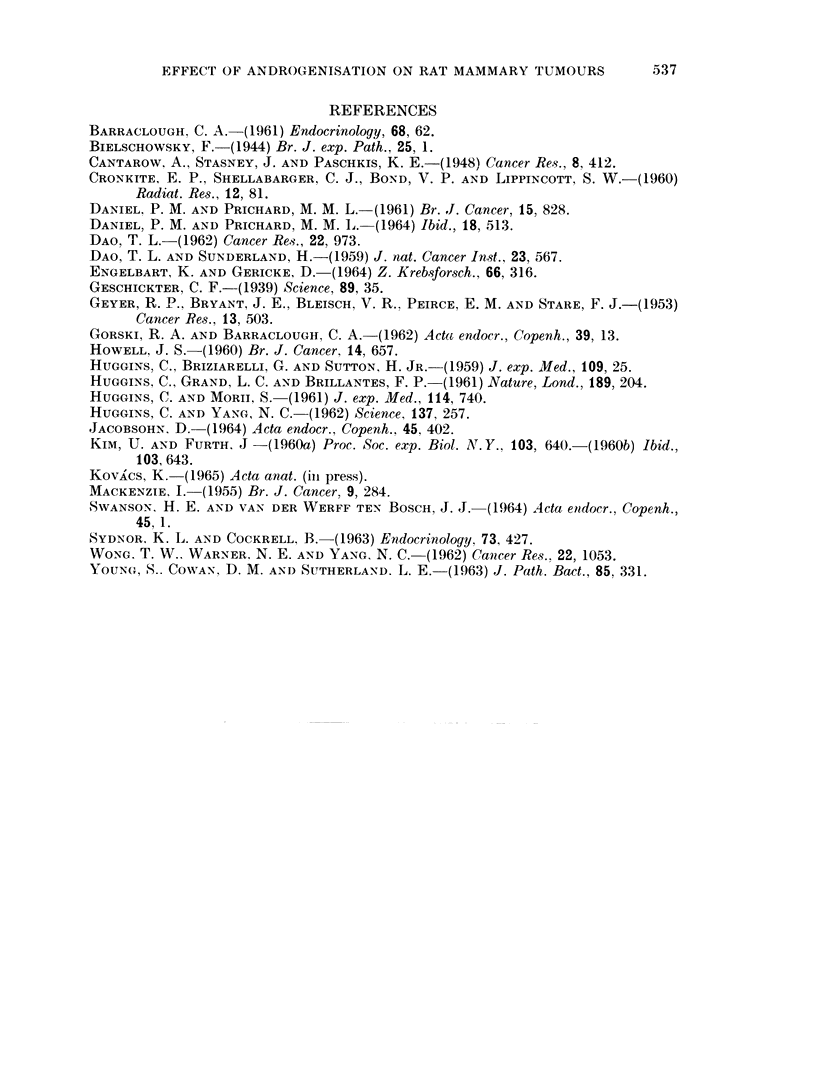# Effect of androgenisation on the development of mammary tumours in rats induced by the oral administration of 9,10-dimethyl-1,2-benzanthracene.

**DOI:** 10.1038/bjc.1965.65

**Published:** 1965-09

**Authors:** K. Kovács

## Abstract

**Images:**


### EFFECT OF ANDROGENISATION ON THE DEVELOPMENT OF MAMMARY TUMOURS IN RATS INDUCED BY THE ORAL ADMINISTRATION OF 9,10-DIMETHYL-1,2-BENZANTHRACENE

K. KOVÁCS

*From the Department of Pathology, University of Liverpool**

Received for publication March 23, 1965

A SINGLE injection of testosterone given to female rats in their early post-natal life results in permanent morphological and functional changes of the gonadotrophin-ovarian axis. The ovaries remain small and though they contain follicles of various sizes, these do not rupture or produce any corpora lutea. Because of the lack of ovulation, these androgenised rats are permanently sterile, and there is a constant cornification of the vaginal epithelium. The luteinizing hormone content of the pituitary is decreased (Barraclough, 1961 ; Gorski and Barraclough, 1962 ; Jacobsohn, 1964 ; Swanson and van der Werff ten Bosch, 1964 ; Kovács, 1965).

As has been shown by Huggins, Grand and Brillantes (1961), Huggins and Yang (1962) and other authors (Dao, 1962 ; Young, Cowan and Sutherland, 1963 ; Daniel and Prichard, 1964) the intragastric administration of 9,10-dimethyl-1,2-benzanthracene (DMBA) to normal female rats results in the development of mammary tumours in a very high percentage of animals within a few months. The present study is concerned with a comparison of the carcinogenic effect of oral administration of DMBA in androgenised and in non-androgenised rats.

### METHODS

Androgenisation was induced when the rats were 1 to 2 days old by a single subcutaneous injection of 2·5 mg. testosterone propionate (Neo-Hombreol, Organon) dissolved in 0·1 ml. oil. When the rats were between 45 and 55 days old, DMBA dissolved in corn oil was administered orally through a gastric tube (a soft rubber urethral catheter). The dose of DMBA will be mentioned later. The animals were carefully palpated for mammary tumours at weekly intervals ; a tumour was recorded only when a nodule at least the size of a small pea could be felt. The animals bearing the tumours died or were killed at various times up to 8 months. The tables show only the number of rats with tumours ; the number and size of the tumours is not presented as these parameters would not provide valid comparisons. The animals were killed with ether and autopsied as soon as possible. The tumours and the various organs were fixed in formol-saline and embedded in paraffin. Sections at 5 $\mu$ were stained with haematoxylin and eosin.

### RESULTS

*Experiment I.*—This experiment was carried out on two groups (non-androgenised and androgenised) of female rats of the Wistar strain. A single oral dose of

* Present address : Department of Medicine, University Medical School, Szeged, Hungary.

50 mg. DMBA dissolved in 2 ml, corn oil was given. The results are shown in Table I.

TABLE I

| Days | Non-androgenised rats: Number of rats | Non-androgenised rats: Rats with mammary tumours | Androgenised rats: Number of rats | Androgenised rats: Rats with mammary tumours |
|---|---|---|---|---|
| 0 | 62 | 0 | 33 | 0 |
| 30 | 23 | 0 | 9 | 0 |
| 60 | 15 | 0 | 7 | 0 |
| 120 | 15 | 0 | 7 | 0 |
| 150 | 15 | 1 | 7 | 0 |
| Extramammary tumour : | | | | |
| colonic adenocarinoma | | 1 | | 0 |

The rats tolerated the 50 mg. of DMBA very badly. Many animals died early ; those that died in the first week or two commonly showed extensive adrenocortical necrosis as described by Huggins and Morii (1961) Dao (1962), and Wong, Warner and Yang (1962). In the non-androgenised rats only one mammary tumour was found and no tumours at all occurred in the androgenised rats. This experiment does not give any information concerning the effect of endocrine factors on the development of mammary tumours, as the number of tumours and the number of surviving animals was too small to allow evaluation of the results. It would appear that the induction of mammary cancers cannot be studied satisfactorily in Wistar rats using a single intragastric administration of 50 mg. DMBA. This dose is too toxic, it is not well tolerated, and tumours of the breast develop only rarely in the few animals which survive over 3 months. This conclusion is in essential agreement with the findings of Engelbart and Gericke (1964).

*Experiment II.*—This experiment was also performed on non-androgenised and androgenised female rats of the Wistar strain. DMBA was administered orally in a dose of 10 mg. in 2 ml. corn oil twice weekly for a period of 3 weeks, so that each rat received a total dose of 60 mg. of DMBA. The results of the experiment are seen in Table II.

TABLE II

| Days | Non-androgenised rats: Number of rats | Non-androgenised rats: Rats with mammary tumours | Androgenised rats: Number of rats | Androgenised rats: Rats with mammary tumours |
|---|---|---|---|---|
| 0 | 48 | 0 | 54 | 0 |
| 50 | 10 | 0 | 24 | 0 |
| 60 | 8 | 1 | 22 | 2 |
| 90 | 6 | 4 | 16 | 5 |
| 120 | 6 | 5 | 16 | 5 |
| 150 | 6 | 6 | 16 | 6 |
| 180 | 6 | 6 | 16 | 8 |
| 210 | 6 | 6 | 16 | 10 |
| 240 | 6 | 6 | 16 | 10 |
| Extramammary tumours : | | | | |
| Stem cell leukaemia | | 4 | | 10 |
| Squamous carcinoma | | 3 | | 3 |
| Renal sarcoma (embryonic type) | | 1 | | 0 |
| Colonic adenocarcinoma | | 1 | | 0 |

Mammary tumours were present in all of the non-androgenised rats which survived for 5 months after the beginning of the DMBA administration. Mammary tumours also developed in the androgenised rats, but the incidence of tumours was less and the time of appearance of the tumours was later.

The repeated small doses of DMBA did not produce any necrosis of the adrenal cortex. However, many of the animals died at 3 to 12 weeks after the first dose of DMBA, and at autopsy it was found that some of them had enlargement of the spleen, liver and lymph nodes. The organs looked very anaemic and petechiae were present on the serous membranes. Histologically the liver and spleen were so infiltrated with immature leucoblastic elements that their architecture was almost totally destroyed (Fig. 1 and 2). Neoplastic foci consisting of primitive white cells were also found occasionally in the renal cortex and in the adrenals (Fig. 3 and 4). The diagnosis of stem cell leukaemia was confirmed by the examination of blood films of some of the animals : the total leucocyte count was elevated and the peripheral blood contained numerous primitive leucoblasts. The occurrence of leukaemia in rats treated orally with DMBA has been previously reported by Dao (1962) and Huggins and Yang (1962). There was no significant difference in the incidence of leukaemia between the non-androgenised and androgenised rats.

Occasionally tumours developed in other organs. These are also listed in Table II. The squamous cell carcinomata arose from the outer ear duct, apparently from Zymbal's gland, a sebaceous gland located periauricularly near the tympanic membrane (Fig. 5). Similar tumours have been described by other authors after oral or intranasal instillation of DMBA (Howell, 1960 ; Huggins, Grand and Brillantes, 1961 ; Huggins and Yang, 1962), after oral administration of methylcholanthrene (Kim and Furth, 1960a) or after painting the skin with an oily solution of DMBA (Howell, 1960).

*Experiment III.*—This experiment was similar to Experiment I, except that Sprague-Dawley rats were used. These were females and in two groups (non-androgenised and androgenised). Each rat received 50 mg. DMBA dissolved in 2 ml. corn oil on one occasion when it was about 50 days old. The results are presented in Table III.

TABLE III

| | Non-androgenised rats | | Androgenised rats | |
|---|---|---|---|---|
| Days | Number of rats | Rats with mammary tumours | Number of rats | Rats with mammary tumours |
| 0 | 60 | 0 | 46 | 0 |
| 50 | 45 | 4 | 34 | 0 |
| 60 | 45 | 6 | 34 | 0 |
| 90 | 43 | 10 | 34 | 1 |
| 120 | 43 | 22 | 34 | 1 |
| 150 | 43 | 31 | 33 | 1 |
| 180 | 43 | 35 | 33 | 2 |
| Extramammary tumours : | | | | |
| Stem cell leukaemia | | 0 | | 3 |
| Squamous carcinoma | | 3 | | 2 |
| Renal sarcoma (embryonic type) | | 1 | | 0 |

The Sprague-Dawley rats tolerated the 50 mg. of DMBA far better than the Wistar rats, though some of them died 1 to 4 weeks after the administration of

DMBA. It can be seen in Table III that mammary tumours developed in a high percentage of the non-androgenised rats, though not as often or as rapidly as reported by Huggins, Grand and Brillantes (1961) and by Huggins and Yang (1962). Nevertheless, it is clear that a single oral administration of 50 mg. DMBA provides a suitable method for the investigation of the importance of endocrine factors in mammary carcinogenesis. It can be seen in Table III that the incidence of the mammary tumours in the androgenised rats is less than in the non-androgenised rats and that the tumours appear later. Some extramammary tumours including stem cell leukaemia also developed in occasional rats. These are also listed in Table III.

As in Experiment I on Wistar rats, extensive adrenocortical necrosis was also found in the Sprague-Dawley rats which died a few days after the oral administration of DMBA. Some animals dying at 1 to 4 weeks showed the same lesion but in a stage of repair with organisation, fibrous replacement and calcification of the necrotic area, and with regeneration of surviving cells in the neighbourhood. In many of the animals which survived over 5 months adrenocortical adenomata were seen ; the exact incidence can not be given as the adrenal glands were not cut in full serial sections. The adenomata were well circumscribed small nodules, hardly visible to the naked eye. They were composed of dark or vacuolated fasciculata cells with cavernous blood vessels and dilated sinusoids filled with blood or eosinophil secretion (Fig. 6).

*Macroscopic and microscopic appearances of the mammary tumours*

The morphological characteristics of the mammary tumours which develop after the oral administration of methylcholanthrene or DMBA have been described by a number of authors (Dao and Sunderland, 1959 ; Huggins, Briziarelli and Sutton, 1959 ; Daniel and Prichard, 1961 ; Huggins, Grand and Brillantes, 1961 ;

---

EXPLANATION OF PLATES

FIG. 1.—Extensive infiltration of the liver by leucoblasts. H. and E. ×260.
FIG. 2.—Numerous leucoblasts in the spleen. H. and E. ×440.
FIG. 3.—Area of leucoblastic infiltration in the renal cortex. H. and E. ×110.
FIG. 4.—Focal leucoblastic infiltration of the adrenal gland. H. and E. ×110.
FIG. 5.—Squamous cell carcinoma originating in Zymbal's gland. H. and E. ×110.
FIG. 6.—Small adenoma of the adrenal cortex composed of dark fasciculata cells. H. and E. ×110.
FIG. 7.—Poorly differentiated adenocarcinoma of the breast. H. and E. ×110.
FIG. 8.—Adenocarcinoma of the breast. The tubules are lined by multiple layers of epithelial cells. H. and E. ×110.
FIG. 9.—Cyst formation in a mammary tumour. H. and E. ×110.
FIG. 10.—A solid area of anaplastic carcinoma of the breast with many mitotic figures. H. and E. ×440.
FIG. 11.—A mammary tumour with a large amount of stroma which is infiltrated with inflammatory cells. H. and E. ×110.
FIG. 12.—Foci of necrosis in an adenocarcinoma of the mammary gland. H. and E. ×110.
FIG. 13.—Milk-secreting adenoma of the mammary gland. H. and E. ×110.
FIG. 14.—Fibroadenoma of the mammary gland. H. and E. ×110.
FIG. 15.—Fibroadenoma of the mammary gland. The fibrous component is well marked. H. and E. ×110.
FIG. 16.—Ovary of an androgenised rat. Follicles of various sizes are seen, but there are no corpora lutea. H. and E. ×36.

1 2 3 4

Kovács.

5

6

7

8

Kovács.

9

10

11

12

13 14 15 16

Kovács.

Young, Cowan and Sutherland, 1963 ; Daniel and Prichard, 1964). The tumours occurring in the present material did not show any marked differences from those found by other authors and a detailed histological description will not be given here.

The tumours developed along the so called milk-line, where breast tissue is normally present. The highest incidence was in the cervical or pectoral regions, but many tumours occurred in the thoracic, abdominal or inguinal regions. Some tumours grew quite rapidly and reached a considerable size, up to 4 or 5 cm. in diameter ; others grew slowly and remained small. The tumours were grossly nodular, well circumscribed and moderately firm. In a few cases the tumours ulcerated. Some rats had a single tumour only, but in several animals, 2, 3 or 4 tumours were present. Metastases were not observed.

Histologically the tumours had the structure of adenocarcinoma with various degrees of differentiation (Fig. 7). Tubules lined by single or multiple layers of epithelial cells were formed, frequently with eosinophil masses of secretion in the lumina (Fig. 8). Papillary growths commonly projected into the lumen, and in many tumours there was a conspicuous formation of cysts (Fig. 9). Sometimes the tumour consisted of solid cell nests. Occasionally the so-called comedo-pattern could be recognised. Some tumours were more anaplastic with numerous mitotic figures (Fig. 10). The quantity of stroma varied between individual tumours. Sometimes it was quite extensive, and was infiltrated to a varying extent with mononuclear inflammatory cells (Fig. 11). Haemorrhages and foci of necrosis were not infrequent (Fig. 12). Microscopic metastases were not seen, but invasion of the neighbouring muscle was common.

In addition so-called milk-secreting adenomata developed in 4 of the non-androgenised Sprague-Dawley rats which had one or two typical mammary tumours as described above and in 1 of the androgenised Sprague-Dawley rats which had no other mammary tumour. Tumours of this type have also been reported by Daniel and Prichard (1964). These tumours consisted of dilated acini lined by a single layer of epithelial cells. The lumina were filled with a milk-like basophilic material. Vacuoles were usually present in this colloid material and also in the cytoplasm of the lining epithelium. The tumours had a histological resemblance to the mammary glands of lactating animals, but there was usually more fibrous stroma and the arrangement of the acini was somewhat irregular (Fig. 13).

Typical fibroadenomata developed in the mammary glands in 7 animals (5 non-androgenised Sprague-Dawley rats and 2 androgenised Sprague-Dawley rats) (Fig. 14 and 15). These tumours are not listed in the tables.

None of these tumours showed any obvious histological difference between the non-androgenised and the androgenised animals.

*Histological changes in the androgenised rats*

In the genital tract of the androgenised rats, typical changes were present identical with those described by previous authors (Barraclough, 1961 ; Jacobsohn, 1964 ; Swanson and van der Werff ten Bosch, 1964 ; Kovács, 1965). The vaginal epithelium was cornified. The ovaries were small and contained follicles of various sizes, some of which showed cystic dilatation. Corpora lutea were not present (Fig. 16).

### DISCUSSION

It is well known that ovarian hormones exert a very important influence on the induction and growth of mammary tumours in experimental animals. Ovariectomy or hypophysectomy markedly reduce the incidence and retard the growth of mammary cancer caused by chemical carcinogens or radiation (Bielschowsky, 1944 ; Huggins, Briziarelli and Sutton, 1959 ; Cronkite, Shellabarger, Bond and Lippincott, 1960 ; Kim and Furth, 1960b ; Dao, 1962 ; Sydnor and Cockrell, 1963 ; Young, Cowan and Sutherland, 1963). Moreover, the administration of oestrogens alone for long periods results in tumour production in the breast of rats (Geschickter, 1939 ; MacKenzie, 1955).

It is very difficult to decide the exact significance of the individual ovarian hormones in the promotion of mammary tumours. Some authors emphasise the importance of oestrogens (Geyer, Bryant, Bleisch, Peirce and Stare, 1953 ; Young, Cowan and Sutherland, 1963). On the other hand numerous findings suggest that progesterone has a more important role (Cantarow, Stasney and Paschkis, 1948; Huggins, Briziarelli and Sutton, 1959 ; Sydnor and Cockrell, 1963).

The present experiments show that the incidence of mammary tumours is definitely reduced in rats which have been androgenised by a single subcutaneous injection of testosterone in early postnatal life. The exact hormonal status of these androgenised rats has not yet been elucidated in detail. Nevertheless it seems clear that they secrete oestrogens but little or no progesterone. If this is so, the present experiments suggest that a deficiency of progesterone has an inhibitory effect on mammary carcinogenesis. It is necessary however to emphasise that this does not rule out the possibility that other endocrine mechanisms may play a part in this effect. For example, there is no information about the rate of luteotrophic hormone secretion in androgenised rats or about the production of androgens by the ovaries in these animals.

The present experiments show further that endocrine stimuli acting in the early postnatal period are able to modify the susceptibility of the rat to carcinogenic factors during later life. Further work is needed to test whether this concept is of general significance.

### SUMMARY

Oral administration of 50 mg. of DMBA to female Sprague-Dawley rats causes mammary cancer in a high percentage of the animals. The incidence of the breast tumours is definitely reduced in androgenised rats, i.e. in rats which have received a single subcutaneous injection of testosterone when they were 1 to 2 days old.

Wistar rats are far less suitable than Sprague-Dawley rats for the study of mammary carcinogenesis induced by DMBA, because they are more susceptible to other toxic effects of the drug. Nevertheless it seems probable that androgenisation reduces the incidence of the mammary tumours in this strain of rat also.

Stem cell leukaemia develops in some of the Wistar and Sprague-Dawley rats treated with DMBA. Androgenisation does not reduce the incidence of the leukaemia.

I am greatly indebted to Professor H. L. Sheehan for his interest help and advice during this study. This work was carried out during the tenure of a Crosby Research Fellowship of the North-West Cancer Research Fund.

### REFERENCES

BARRACLOUGH, C. A.—(1961) *Endocrinology*, **68**, 62.
BIELSCHOWSKY, F.—(1944) *Br. J. exp. Path.*, **25**, 1.
CANTAROW, A., STASNEY, J. AND PASCHKIS, K. E.—(1948) *Cancer Res.*, **8**, 412.
CRONKITE, E. P., SHELLABARGER, C. J., BOND, V. P. AND LIPPINCOTT, S. W.—(1960) *Radiat. Res.*, **12**, 81.
DANIEL, P. M. AND PRICHARD, M. M. L.—(1961) *Br. J. Cancer*, **15**, 828.
DANIEL, P. M. AND PRICHARD, M. M. L.—(1964) *Ibid.*, **18**, 513.
DAO, T. L.—(1962) *Cancer Res.*, **22**, 973.
DAO, T. L. AND SUNDERLAND, H.—(1959) *J. nat. Cancer Inst.*, **23**, 567.
ENGELBART, K. AND GERICKE, D.—(1964) *Z. Krebsforsch.*, **66**, 316.
GESCHICKTER, C. F.—(1939) *Science*, **89**, 35.
GEYER, R. P., BRYANT, J. E., BLEISCH, V. R., PEIRCE, E. M. AND STARE, F. J.—(1953) *Cancer Res.*, **13**, 503.
GORSKI, R. A. AND BARRACLOUGH, C. A.—(1962) *Acta endocr., Copenh.*, **39**, 13.
HOWELL, J. S.—(1960) *Br. J. Cancer*, **14**, 657.
HUGGINS, C., BRIZIARELLI, G. AND SUTTON, H. JR.—(1959) *J. exp. Med.*, **109**, 25.
HUGGINS, C., GRAND, L. C. AND BRILLANTES, F. P.—(1961) *Nature, Lond.*, **189**, 204.
HUGGINS, C. AND MORII, S.—(1961) *J. exp. Med.*, **114**, 740.
HUGGINS, C. AND YANG, N. C.—(1962) *Science*, **137**, 257.
JACOBSOHN, D.—(1964) *Acta endocr., Copenh.*, **45**, 402.
KIM, U. AND FURTH, J—(1960*a*) *Proc. Soc. exp. Biol. N.Y.*, **103**, 640.—(1960*b*) *Ibid.*, **103**, 643.
KOVÁCS, K.—(1965) *Acta anat.* (in press).
MACKENZIE, I.—(1955) *Br. J. Cancer*, **9**, 284.
SWANSON, H. E. AND VAN DER WERFF TEN BOSCH, J. J.—(1964) *Acta endocr., Copenh.*, **45**, 1.
SYDNOR, K. L. AND COCKRELL, B.—(1963) *Endocrinology*, **73**, 427.
WONG, T. W., WARNER, N. E. AND YANG, N. C.—(1962) *Cancer Res.*, **22**, 1053.
YOUNG, S., COWAN, D. M. AND SUTHERLAND, L. E.—(1963) *J. Path. Bact.*, **85**, 331.